# Data and video for the thermal and fire propagation of multiple lithium-ion batteries

**DOI:** 10.1016/j.dib.2019.104379

**Published:** 2019-08-28

**Authors:** Mingyi Chen, Dongxu Ouyang, Jiahao Liu, Jian Wang

**Affiliations:** a School of the Environment and Safety Engineering, Jiangsu University, Zhenjiang, 212013, Jiangsu, China; b Firefighting and Rescue Technology Key Laboratory of the Ministry of Public Security, China People's Police University, Langfang, 065000, Hebei, China; c State Key Laboratory of Fire Science, University of Science and Technology of China, Hefei, 230026, Anhui, China; d College of Ocean Science and Engineering, Shanghai Maritime University, Shanghai, 201306, China

**Keywords:** Lithium-ion battery, Pack, Thermal behavior, Fire propagation

## Abstract

The data presented in this article are related to research article “Investigation on thermal and fire propagation behaviors of multiple lithium-ion batteries within the package” (Chen et al., 2019). This data article provides the data information including the experiment pictures, flame temperatures, pressure and heat flux sensors temperatures, and gas concentrations of 6 × 6 batteries and 10 × 10 batteries. The video of the whole thermal and fire propagation behaviors of 6 × 6 batteries is also provided.

**Table:** Specifications Table

Subject	Engineering
Specific subject area	Safety engineering
Type of data	Image Graph Figure Video
How data were acquired	Camera, Thermocouples, Servomex 4100 gas analyser,
Data format	Raw
Parameters for data collection	The collection of temperature and gas concentration data is mainly in the case of thermal runaway and fire in the battery pack.
Description of data collection	Temperature data is collected by thermocouples. Gas concentration data was collected by Servomex 4100 gas analyser. Video and image data is recorded by the camera.
Data source location	University of Science and Technology of China, Hefei, China
Data accessibility	With the article
Related research article	Mingyi Chen, Ouyang Dongxu, Jiahao Liu, Jian Wang, Investigation on thermal and fire propagation behaviors of multiple lithium-ion batteries within the package, Applied Thermal Engineering DOI 10.1016/j.applthermaleng.2019.113750

**Table:** 

**Value of the data** • The experiment pictures can be a reference for the further lithium-ion battery fire studies. • The video presented clearly demonstrate the fire propagation behaviors of multiple lithium-ion batteries. • Lithium-ion battery fire can be more intuitively understanding form the flame temperatures.

## 1 Data

**Fig. 1:**
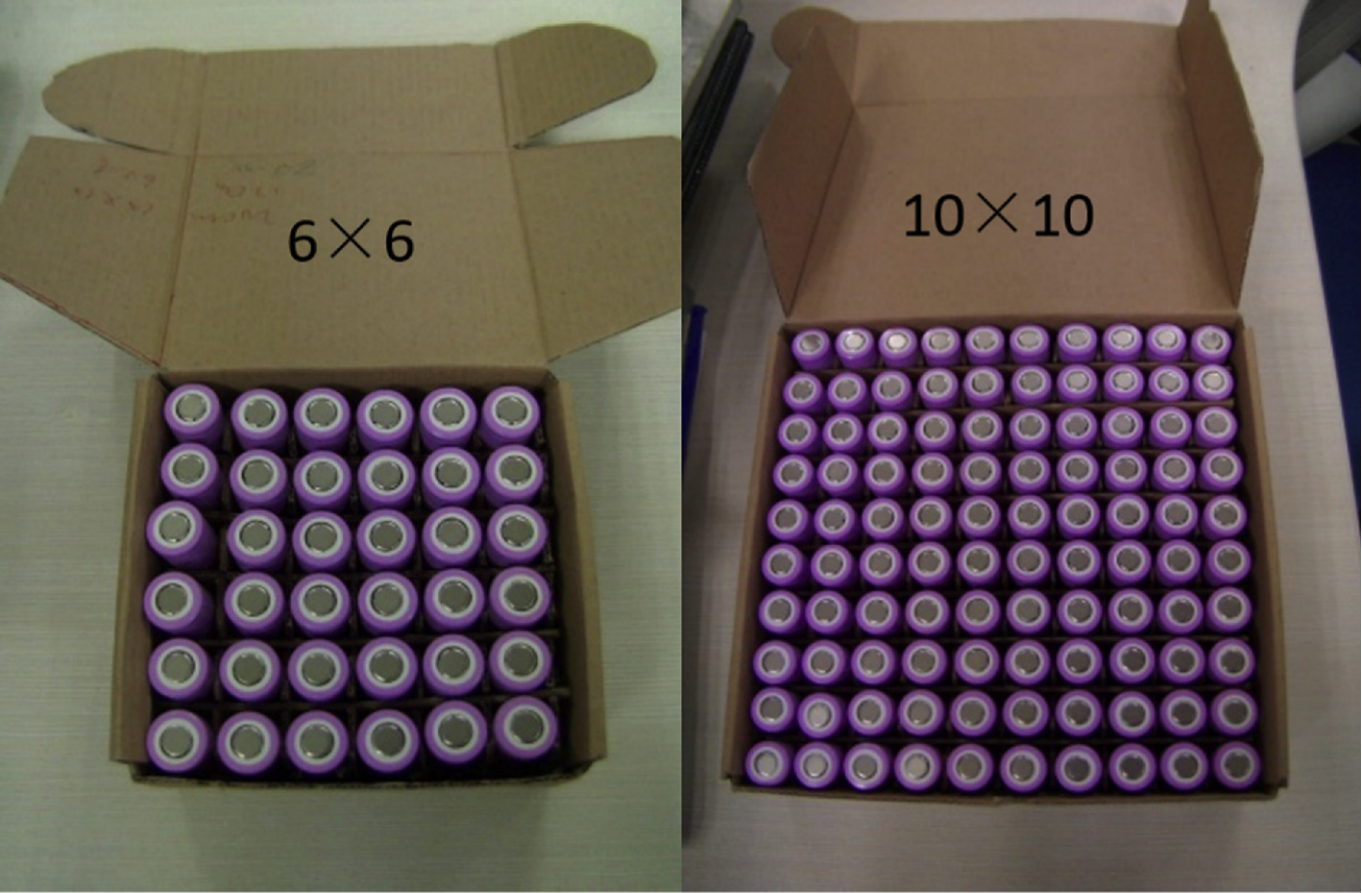
**Structure of the batteries inside the cardboard box**. The batteries are SAMSUNG 18650 type LiCoO_2_ cells which are charged to 80% SOC (commonly used state for storage and transportation). The batteries were packed in a cardboard box supplied by the manufacturer, and each cell was separated by crossed hard papers.

**Fig. 2:**
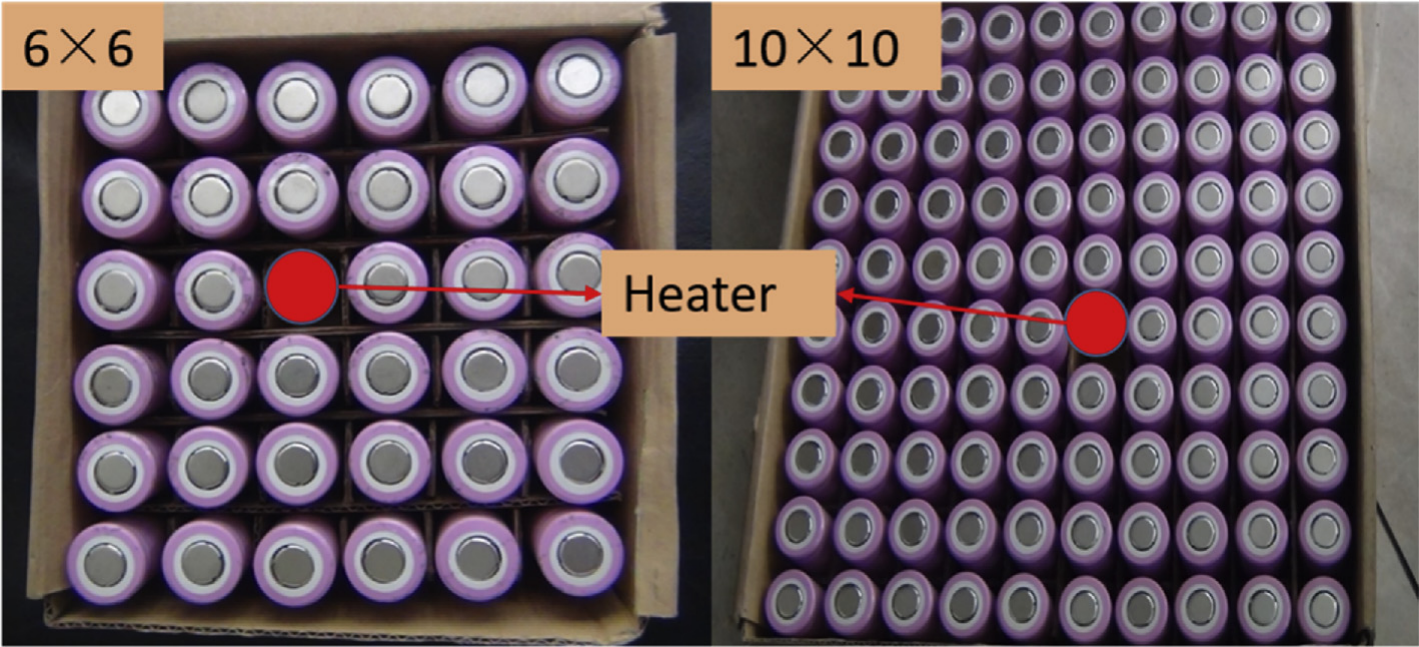
**Position of the heater in the pack**. The red circle here simply represents the heating rod. The middle of the cardboard box was hollowed out, and a 500 W heating rod with similar dimensions of the battery was used to achieve the purpose of thermal propagation.

**Fig. 3:**
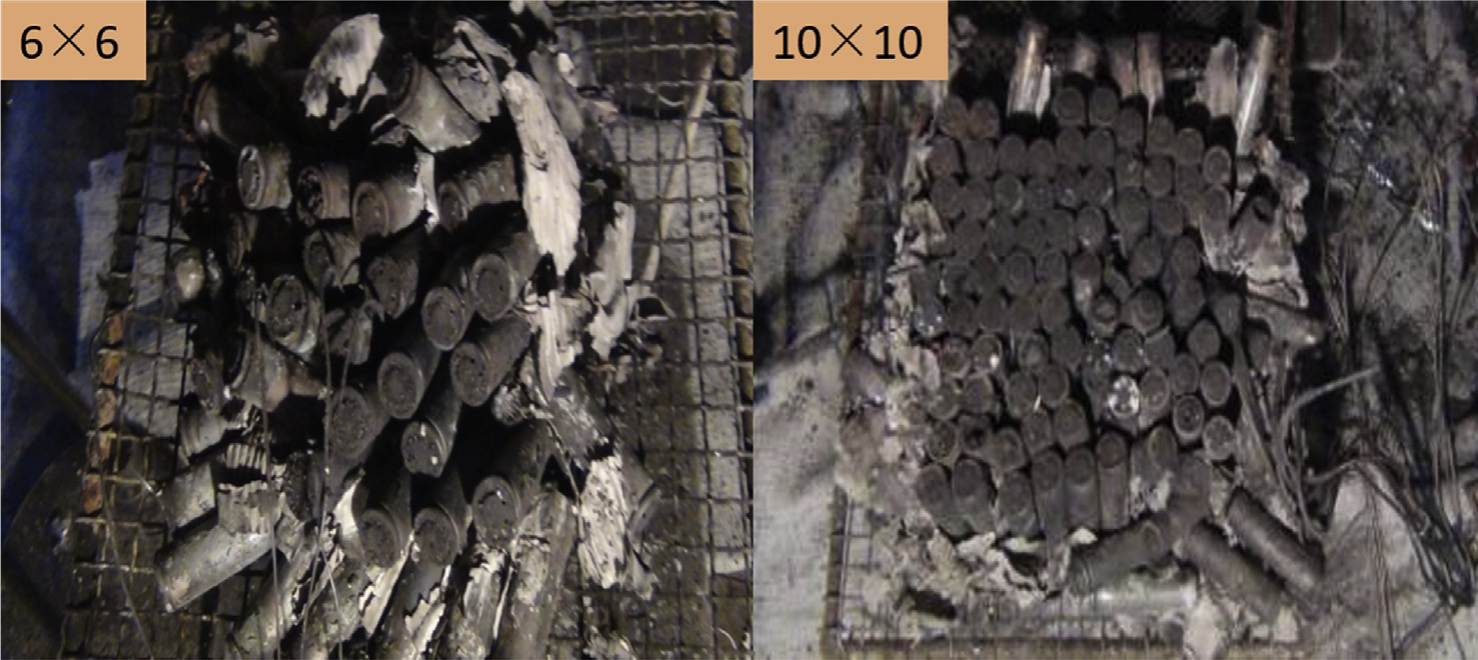
**Burned batteries after the fire tests**. At the end of the combustion experiment, the shape of the burning residue of the battery pack was photographed with a camera. The shooting angle is directly above the battery pack. The state of each battery in the battery pack is original after the end of the experiment, and there is no manual disturbance.

**Fig. 4:**
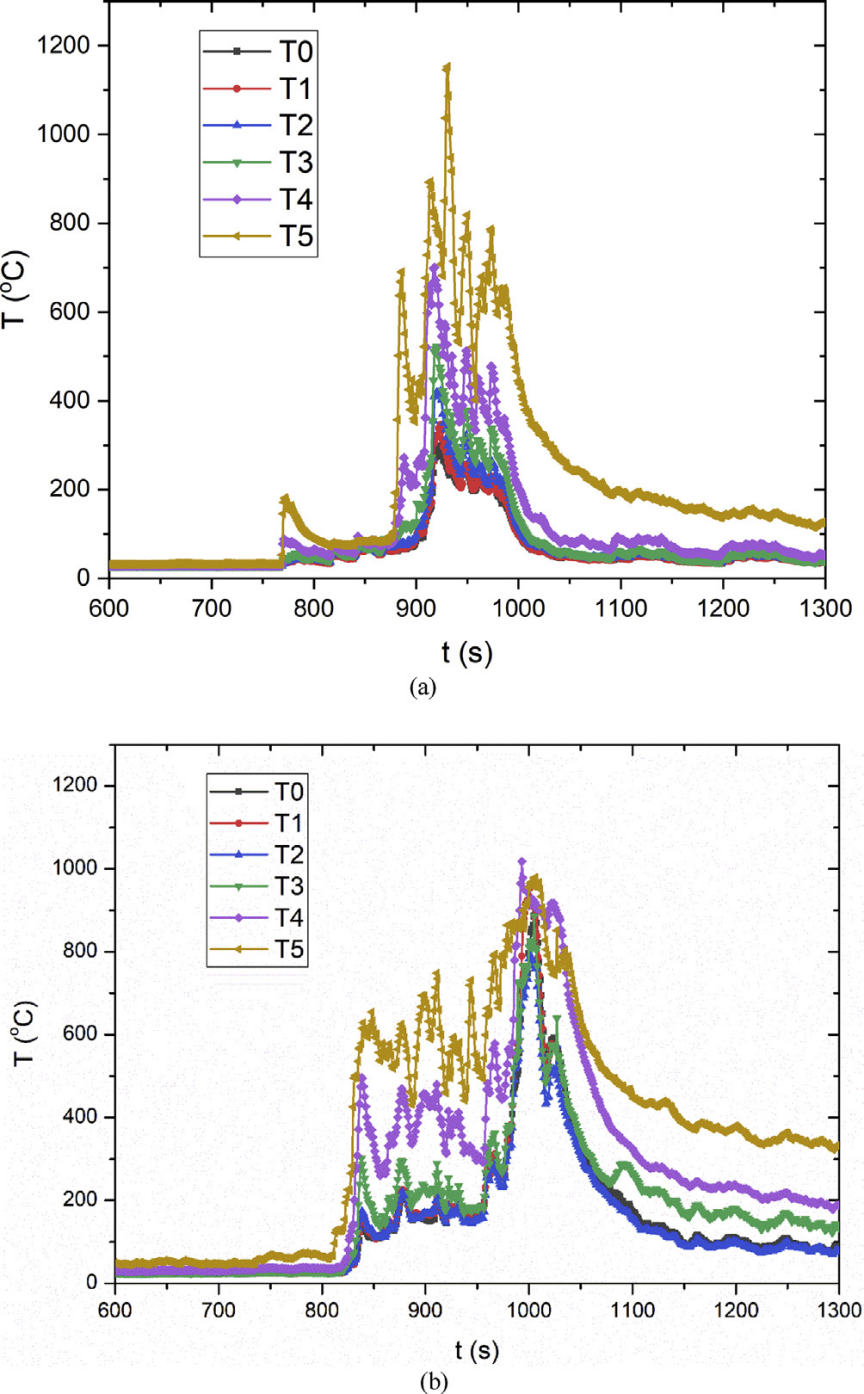
**Flame temperatures**. Flame temperatures of (a) 6 × 6 batteries and (b) 10 × 10 batteries during the thermal and fire propagation. The abscissa is time and the ordinate is temperature change. Since the combustion development period is relatively concentrated, the abscissa has only taken 600–1300 seconds.

**Fig. 5:**
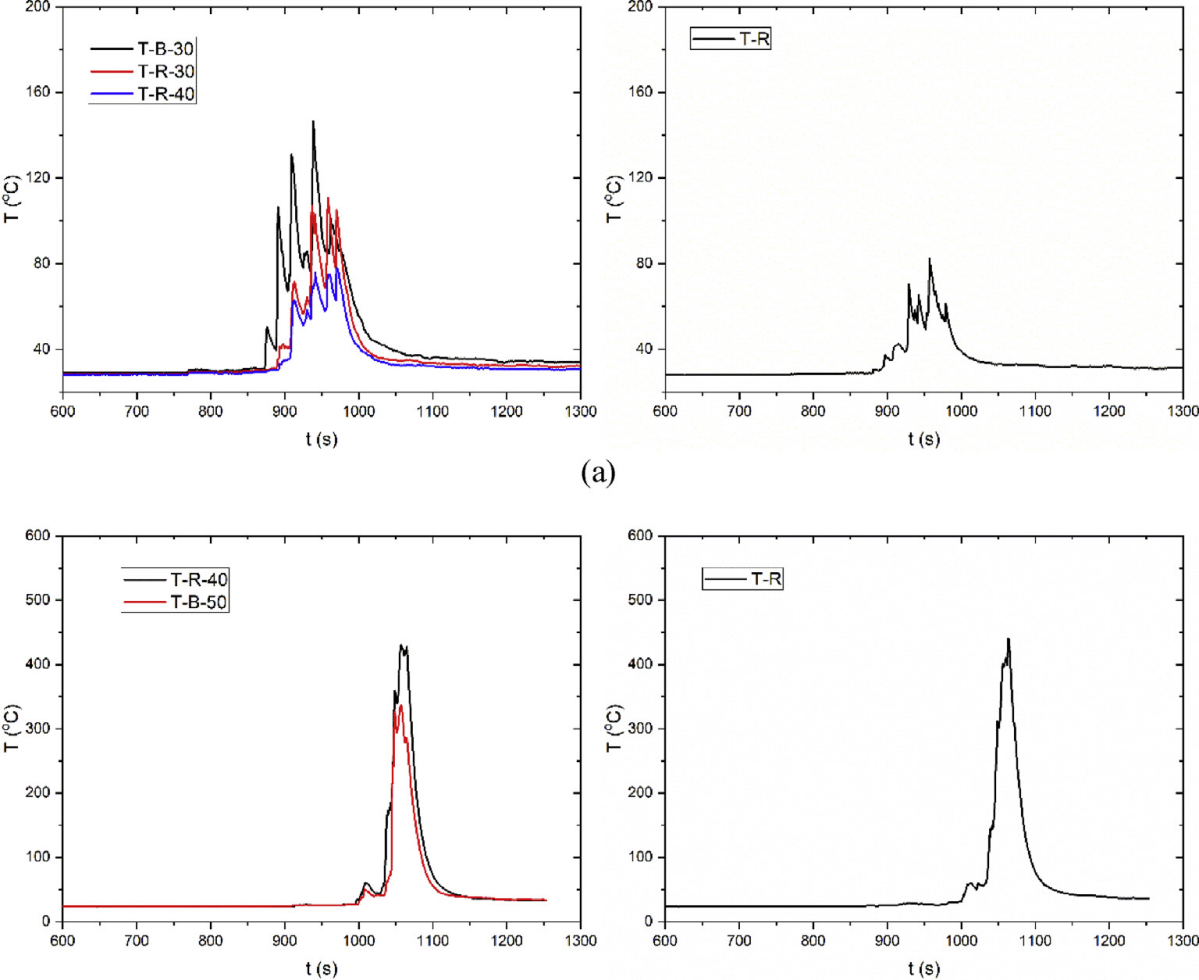
**Pressure and heat flux sensors temperatures**. Pressure and heat flux sensors temperatures of (a) 6 × 6 batteries and (b) 10 × 10 batteries during the thermal and fire propagation. B stands for the rear side position and R stands for the right side position. 30 and 40 represent the distance of the sensor from the center of the battery pack.

**Fig. 6:**
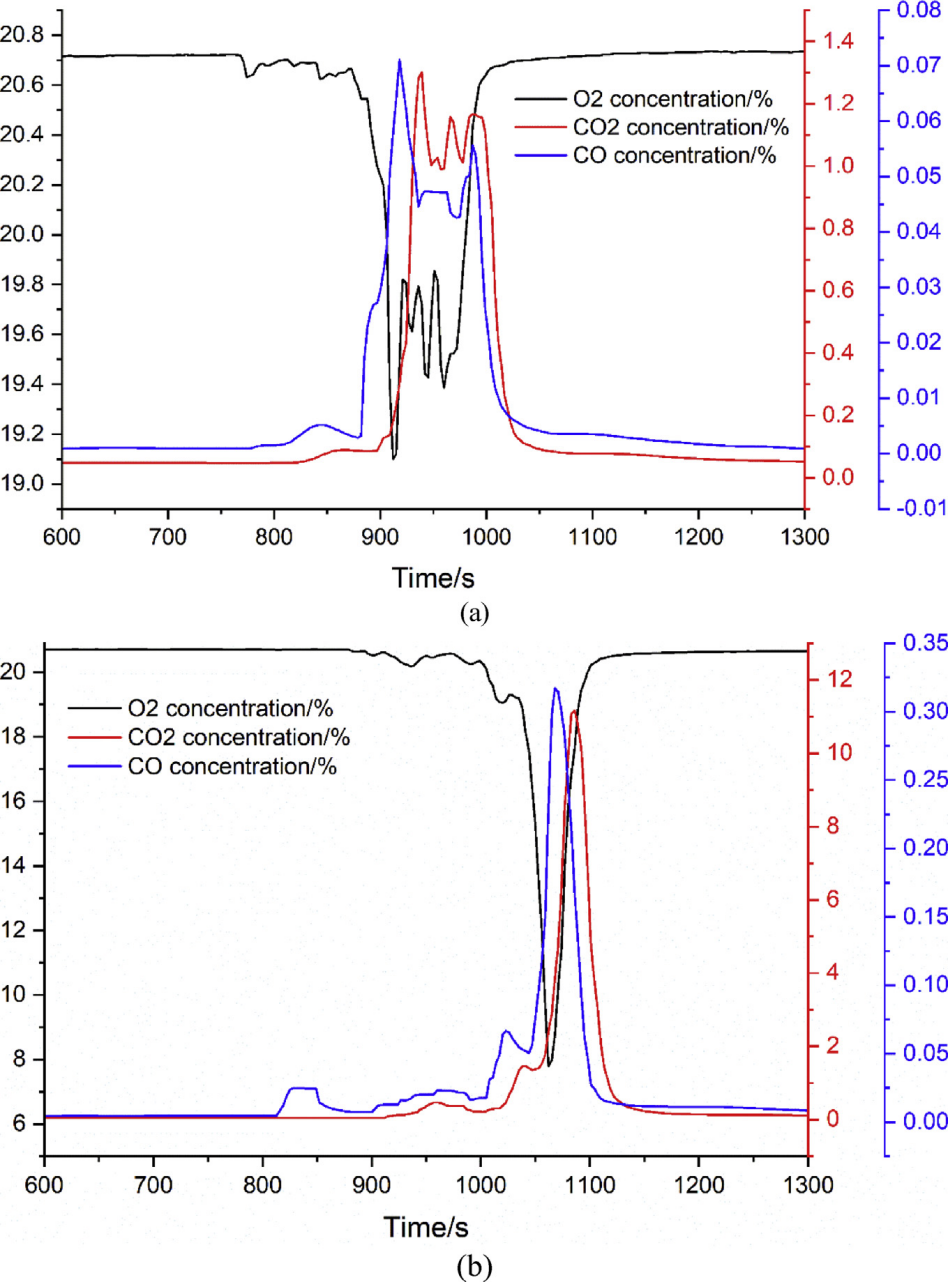
**O**_**2**_**, CO**_**2**_**, and CO concentration changes**. Oxygen, carbon dioxide, and carbon monoxide concentration changes of (a) 6 × 6 batteries and (b) 10 × 10 batteries during the thermal and fire propagation. The black ordinate is the axis of oxygen concentration change. The red ordinate is the axis of carbon dioxide concentration change. The blue ordinate is the axis of carbon monoxide concentration change. The gas concentration unit is the volume fraction in air and expressed as percentage.

The dataset in this article describes the thermal and fire propagation process of multiple lithium-ion batteries with varying pack scale. Fig. 1 presents the constructions of the battery pack for 6 × 6 batteries and 10 × 10 batteries. Fig. 2 shows the position of the heater in the pack. Fig. 3 presents the batteries which are completely burned. Video 1 shows the whole thermal and fire propagation process for 6 × 6 batteries. Fig. 4 shows the flame temperatures changes. Fig. 5 shows the pressure and heat flux sensors temperatures. Fig. 6 shows oxygen, carbon dioxide, and carbon monoxide concentration changes.

Supplementary video related to this article can be found at https://doi.org/10.1016/j.dib.2019.104379

The following is the supplementary data related to this article:

Video 1 1 Video 1

## 2 Experimental design, materials, and methods

Experiments were carried out to study the thermal changes and fire behaviors during the thermal and fire propagation process of multiple lithium-ion batteries as detailed in Ref [1]. This article provides the data for a better understanding of the fire behaviors and the pictures and video are mainly obtained by means of camera recording.

**Fig. 7:**
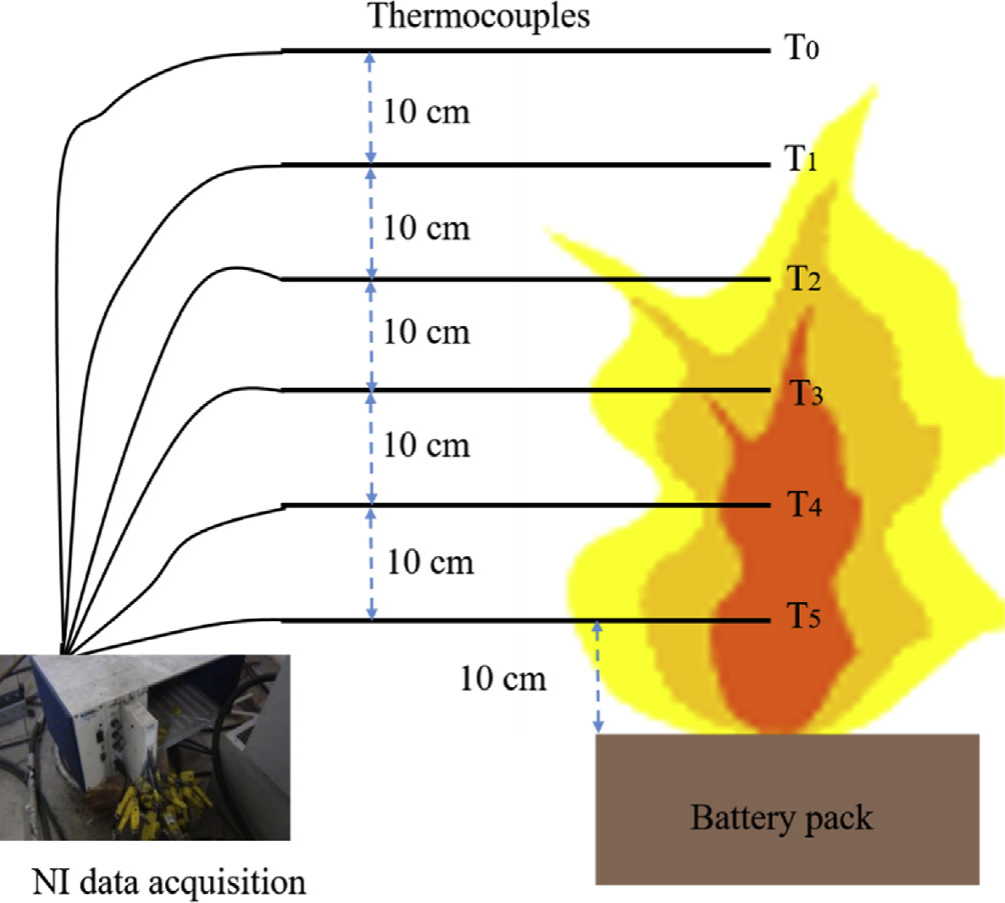
Experiment setup for flame temperatures measurement.

Flame temperature is an important parameter for analyzing fire hazards. A plurality of thermocouples are placed above the battery pack to measure the flame temperatures of multiple lithium-ion batteries fire. The thermocouples are arranged as shown in Fig. 7. The thermocouples were placed every 10 cm above the center of the battery pack, and a total of six thermocouples were set. To measure the flame temperatures of the lithium-ion battery fire, the same thermocouples as in Ref [1] were used. The thermocouples are K-type with 1 mm diameter, 1400 K measurement range, and 0.5 K precision. National Instruments (NI) data acquisition instruments are used to record temperature data transmitted by thermocouples.

The ambient temperature at which the pressure and heat flux sensors probes are located is an important parameter for examining their operating conditions. The positions of the pressure and heat flux sensors probes are specifically described in Ref [1].

Three simultaneous gas stream measurements of oxygen, carbon dioxide, and carbon monoxide are analyzed in the Servomex 4100 gas analyzer.
